# Analysis platform for hemodynamic function in congenital heart disease

**DOI:** 10.1186/1532-429X-11-S1-P126

**Published:** 2009-01-28

**Authors:** Elizabeth J Nett, Kevin M Johnson, Christopher J Francois, Oliver Wieben

**Affiliations:** University of Wisconsin, Madison, WI USA

**Keywords:** Congenital Heart Disease, Wall Shear Stress, Flow Measurement, Respiratory Gating, Complex Congenital Heart Disease

## Introduction

Advanced Phase Contrast MR acquisition techniques such as PC VIPR [1] can simultaneously provide anatomical images and cine velocity fields with volumetric coverage. These data can be used for non-contrast enhanced MR Angiography, velocity and flow measurements, and the derivation of addition hemodynamic parameters such as trans-stenotic pressure gradients and wall shear stress. While this comprehensive information can be very valuable, particularly in complex congenital heart disease, the large data volume with high spatial and temporal information requires new approaches for efficient visualization and extraction of clinically relevant parameters.

## Purpose

To develop a software platform that streamlines hemodynamic analysis including flow measurements with automatic alignment with respect to the vessel orientation and the derivation of hemodynamic parameters such as wall shear stress and pressure gradients.

## Methods

**Figure 1:**
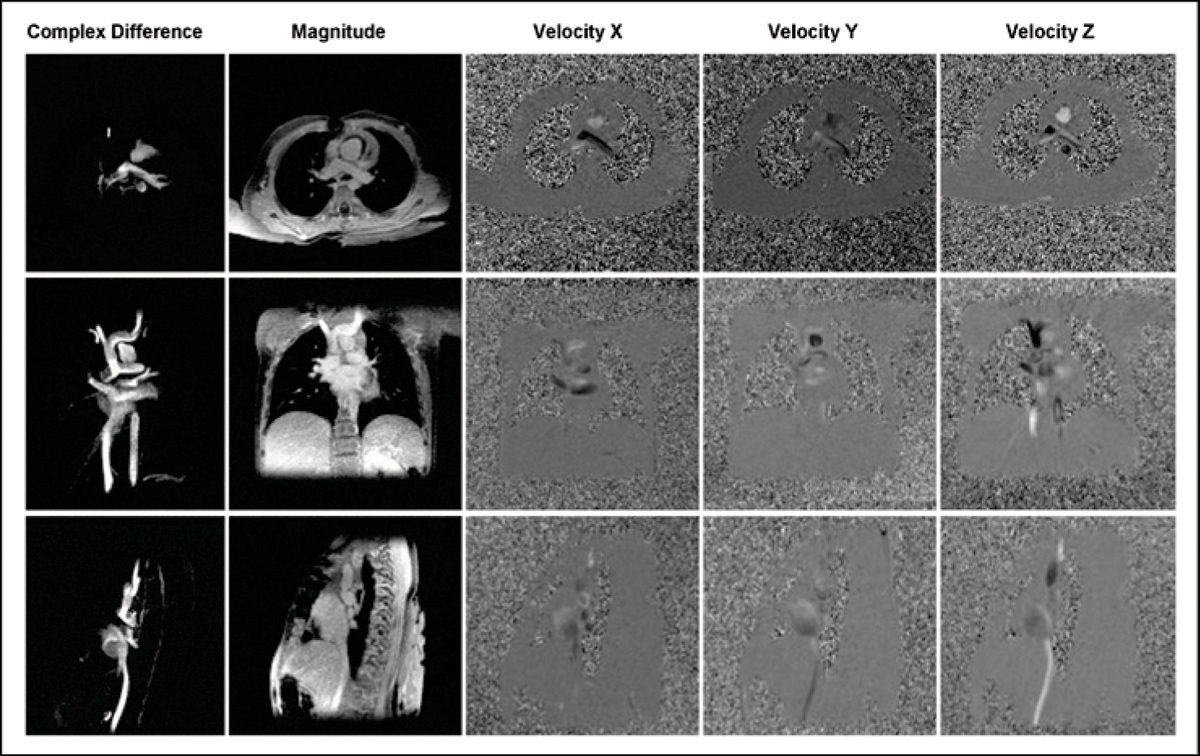
**Time averaged complex difference, magnitude and velocity images in three orthogonal directions in the axial, saggital and coronal slices from a PC VIPR data set of a patient with pulmonary venolobar sydrome**. The large data volume requires a new, efficient approach to data processing and visualization.

Data were acquired with a dual-echo PC VIPR trajectory with cardiac and respiratory gating on 1.5 T and 3 T clinical systems (GE Healthcare) [2]. Typical scan parameters were: (1.0–1.25 mm)^3^ isotropic spatial resolution in approximately 10 min scan time with 50% respiratory gating efficiency, imaging volume: 32 × 32 × 16 cm^3^, VENC of 50–100 cm/s (application specific). Figure 1 displays representative axial, coronal, and sagittal reformats of the magnitude, three velocity components, and the derived angiogram. These data sets contain 320 slices × 20 time frames × 5 volumes = 32,000 images.

We developed a software platform with a visual interface using Matlab (MathWorks) in order to visualize the results, enable quantitative measurements, and derive additional hemodynamic parameters. First, the reconstructed PC VIPR data are loaded into a segmentation tool in order to reduce the size of the data set for more efficient processing. The knowledge of vessel boundaries is also essential for the calculations of flow, wall shear stress, and pressure gradients, which are available processing tools. Next the data can be loaded into the analysis plug-ins to measure and visualize flow, derive pressure maps [3], or calculate wall shear stress [4] from the velocity fields with volumetric coverage. For improved flow measurements, the analysis plane can be automatically aligned perpendicular to the vessel path. Flow measurements are derived by integration of the velocity vectors over time and vessel area, which can be defined either automatically with a threshold algorithm or manual selection. Velocity and flow measurements as well as the time average and/or cine volumetric maps of pressure gradients and wall shear stress can be exported for further analysis and visualization or comparisons with computational fluid dynamics (CFD) calculations. To validate the velocity and flow measurements, multiple PC VIPR data sets including flow phantom data and in vivo cardiac data were compared with standard 2D PC MR measurements prescribed perpendicular to the vessel orientation.

## Results

**Figure 2:**
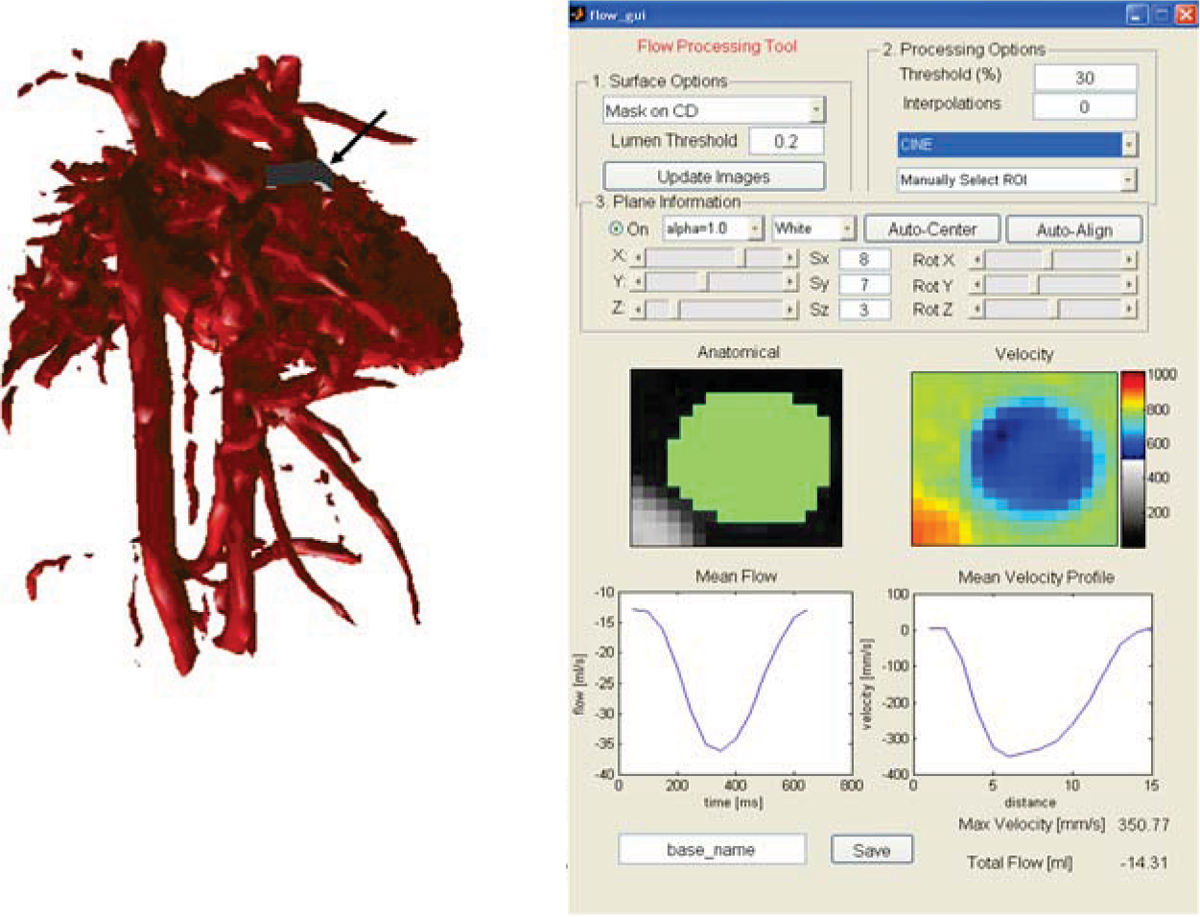
**Matlab flow analysis software used on a PC VIPR data set of a patient with Scimitar syndrome**. An arrow points to the ROI box (grey) that can be automatically centered and aligned with the vessel and the through plane velocities. Based on the RIO selection, the flow through the cardiac cycle and profile of the mean velocity can be derived.

Figure 2 shows the flow and velocity tool used in a patient with Scimitar syndrome. Among other parameters, the through-plane flow of the analyzed vessel segment is displayed as a function of time in the cardiac cycle and as net flow in addition to a velocity profile across the vessel diameter. For the validation of the flow measurements, good agreement was found between PC VIPR and 2D PC measurements.

## Conclusion

A new software platform was implemented for the comprehensive analysis of hemodynamic information obtainable from high resolution PC MR datasets. Future studies using this platform will help to identify the clinically significant hemodynamic parameters for clinical applications such as early diagnosis, treatment monitoring, and understanding of disease development in congenital heart disease.
